# Effects of Genic Base Composition on Growth Rate in G+C-rich Genomes

**DOI:** 10.1534/g3.115.016824

**Published:** 2015-04-20

**Authors:** Yogeshwar D. Kelkar, Daniel S. Phillips, Howard Ochman

**Affiliations:** Department of Ecology and Evolutionary Biology, Yale University, New Haven, Connecticut 06520

**Keywords:** base composition, natural selection, mutational patterns, microbial genomes

## Abstract

The source and significance of the wide variation in the genomic base composition of bacteria have been a matter of continued debate. Although the variation was originally attributed to a strictly neutral process, *i.e.*, species-specific differences in mutational patterns, recent genomic comparisons have shown that bacteria with G+C-rich genomes experience a mutational bias toward A+T. This difference between the mutational input to a genome and its overall base composition suggests the action of natural selection. Here, we examine if selection acts on G+C contents in *Caulobacter crescentus* and *Pseudomonas aeruginosa*, which both have very G+C-rich genomes, by testing whether the expression of gene variants that differ only in their base compositions at synonymous sites affects cellular growth rates. In *C. crescentus*, expression of the more A+T-rich gene variants decelerated growth, indicating that selection on genic base composition is, in part, responsible for the high G+C content of this genome. In contrast, no comparable effect was observed in *P. aeruginosa*, which has similarly high genome G+C contents. Selection for increased genic G+C-contents in *C. crescentus* acts independently of the species-specific codon usage pattern and represents an additional selective force operating in bacterial genomes.

Base composition is highly variable among bacterial species, which ranges among sequenced genomes from 13% to 75% G+C (McCutcheon and Moran 2010; Thomas *et al.* 2008). Several bacterial phyla contain both high and low G+C lineages, suggesting to some that the repeated, independent emergence of this variation results from a selective force that favors certain base compositions under particular environment conditions (McEwan *et al.* 1998; Naya *et al.* 2002; Rocha and Danchin 2002; Romero *et al.* 2009). A contrasting interpretation, posited more than 50 years ago by both Sueoka (1962) and Freese (1962), explained the diversity in genomic base compositions among bacteria in terms of a neutral model in which the variation was attributed to the inherent differences among species in their patterns of mutations.

This decades-old view that variation in genomic base composition is neutral and driven by differences in the mutational process has been bolstered by: (i) the inability to ascertain the selective agent that acts on base composition (Basak and Ghosh 2005; Bentley and Parkhill 2004; Bohlin *et al.* 2010; Galtier and Lobry 1997; Hurst and Merchant 2001; Rocha and Feil 2010; Wang *et al.* 2006); (ii) the unrealistic requirements about the strength of selection necessary to favor a single base compositional change in a genome (Rocha and Feil 2010); and (iii) the recognition that certain DNA replication and repair enzymes alter genomic G+C contents (Cox and Yanofsky 1967; Lind and Andersson 2008). However, recent comparisons of sequenced genes and genomes have shown that the mutational pattern in bacteria is biased toward A and T, especially in those genomes that are G+C-rich (Hershberg and Petrov 2010; Hildebrand *et al.* 2010; Van Leuven and McCutcheon 2012). The discrepancy between the mutational input and the observed base composition counters the neutral view and suggests that natural selection, or another mechanism such as biased gene conversion (Duret and Galtier 2009; Touchon *et al.* 2009; Birdsell 2002; Lassalle *et al.* 2015), shapes the nucleotide compositions of bacterial genomes—specifically, that there is preference for increased G+C content in the majority of the sequenced bacterial genomes.

To date, virtually every study that has searched for the underlying basis of the compositional variation among bacteria has been retrospective, based chiefly on the relationship between genomic G+C contents and some environmental variable or molecular feature (Rocha and Feil 2010). Given the evidence from sequence comparisons that selection serves to increase G+C contents in G+C-rich genomes, we adopted an experimental approach to test the effects of altering base compositions by assaying *E. coli* strains expressing genes of different G+C contents. Those strains expressing G+C-rich versions of genes displayed higher growth rates than those expressing the identical protein from A+T-rich versions (Raghavan *et al.* 2012), showing that selection on base composition is occurring at the level of the gene. These results are supported by the observation that four-fold degenerate sites often have more extreme base composition than any intergenic regions, although both classes of sites are traditionally assumed to be evolving neutrally to the same base composition (Muto and Osawa 1987; Hershberg and Petrov 2010; Raghavan *et al.* 2012).

If selection on the G+C richness of genes is the source of the base compositional variation in bacterial genomes, it should be most evident in species whose genomes have very high G+C contents. Therefore, we examined whether gene-level selection on base composition, similar to that observed in *E. coli*, is operating in two phylogenetically divergent species, *Caulobacter cresentus* and *Pseudomonas aeruginosa*. These species have among the highest G+C contents of any bacterial genome (67% G+C) and are therefore expected to show some of the most pronounced effects of selection for increased G+C contents.

## Materials and Methods

### Bacterial strains and plasmids

*Caulobacter crescentus* CB15 (NA1000) and *Pseudomonas aeruginosa* PAO1 were used as hosts to express variants of the green fluorescent protein (GFP) gene that encoded the identical protein but differed in base composition at synonymous sites. For expression in *C. crescentus*, GFP variants were cloned into pBXMCS-2, which confers kanamycin resistance and carries a xylose inducible promoter (Pxyl). For expression in *P. aeruginosa*, GFP variants were cloned into pNW33N, which confers chloramphenicol resistance and carries the IPTG-inducible Phyperspank promoter (Mirończuk *et al.* 2008).

### Plasmid constructs

GFP variants were each amplified from their original pET15 expression vectors (Raghavan *et al.* 2012) using primers that included restriction sites to enable cloning into the appropriate vector. For cloning into pBXMCS-2, the primers incorporated *Apa*I and *Eco*R1 restriction sites, which placed the GFP gene downstream to the Pxyl promoter. For cloning into pNW33N, the primers incorporated restriction sites for *Bpu*10I and *Bzt*Z17I. Primer sequences are listed in Supporting Information, Table S1. Digestions and ligations proceeded according to the supplier’s instructions (NEB), and the identity of all constructs was confirmed by sequencing. These plasmids as well as plasmids lacking any GFP insert were transformed into the appropriate bacterial host by electroporation. A total of 12 GFP variants were placed into the *C. crescentus* background. Nine were placed in the *P. aeruginosa* background. The GFP variants ranged from 40.4% to 55.7% G+C and differed only in their base compositions at synonymous sites.

### Fitness assays

To test the effects of GFP expression on the growth rate of *C. crescentus*, 3 μl of an overnight culture of each strain containing a GFP variant were inoculated into 147 μl of Peptone-Yeast Extract medium supplemented with 5 μg/ml kanamycin. After propagation for 2 hr at 30°, xylose was added to a final concentration of 0.03% to induce GFP expression. The optical density (OD_600_) and GFP fluorescence of each culture were measured every hour for 10 hr on a Victor3 Microplate Reader (PerkinElmer). Growth rate experiments in *P. aeruginosa* proceeded similarly except that: (1) cells were propagated in LB containing 17 μg/ml chloramphenicol at 37°; (2) 1 mM IPTG was added to induce GFP expression; and (3) fluorescence and optical density were measured every hour for 4 hr. To gauge GFP expression, fluorescence values of cultures were normalized using the fluorescence values of negative controls, *i.e.*, cultures harboring no GFP inserts, as

$$Fn_{GFPi}=F_{GFPi}-\left(\frac{OD600_{GFPi}*F_{CONTROL}}{OD600_{CONTROL}}\right)$$

where *F_GFPi_* and *OD600_GFPi_* are the observed fluorescence and absorbance values, respectively, of cultures with GFP clone *i*, *Fn_GFPi_*, the normalized fluorescence of the culture, and *F_CONTROL_* and *OD600_CONTROL_* are the observed fluorescence and absorbance values, respectively, of the negative control. Measurements of optical density and fluorescence of the growth cultures are provided in Table S2 (*C. crescentus*) and Table S3 (*P. aeruginosa*).

### Computational analysis

The codon adaptation index (CAI) of each GFP variant was calculated using the method of Sharp and Li (1987) using EMBOSS Explorer (embossgui.sourceforge.net) as follows. For each species, a reference set of genes encoding highly expressed proteins (ribosomal proteins, elongation factors, and chaperonins) was used to compile codon usage table using the “cusp” tool. These codon usage tables were used by the “cai” tool to calculate the species-specific CAI values. Complete genome sequences were obtained from Genbank. Shine-Dalgarno (SD)-like sequences that have high predicted affinity (>6 kcal/mol) to anti-SD sites of bacterial ribosomes were obtained (Li *et al.* 2012) and their frequencies in each GFP variant measured. Regression analyses were performed using the lm package in the R statistical environment (R Development Core Team 2011).

## Results

Comparative sequence analysis suggested a role for selection in increasing the G+C contents of protein-coding genes in virtually all G+C-rich bacterial genomes (Hershberg and Petrov 2010; Hildebrand *et al.* 2010). We tested this hypothesis experimentally in *Caulobacter crescentus* and *Pseudomonas aeruginosa*, which have among the highest genomic G+C contents of any bacterial species. Specifically, in each species, we assayed the growth rates of isogenic strains that gratuitously expressed variants of a GFP reporter gene that each differed in its G+C content at synonymous sites, with the goal of establishing whether selection is actually acting on the base composition of genes, not genomes *per se*. Moreover, use of the same GFP variants as those in a previous study (Raghavan *et al.* 2012) allowed us to better define the manner in which selection is operating.

### Caulobacter crescentus

**Figure 1:**
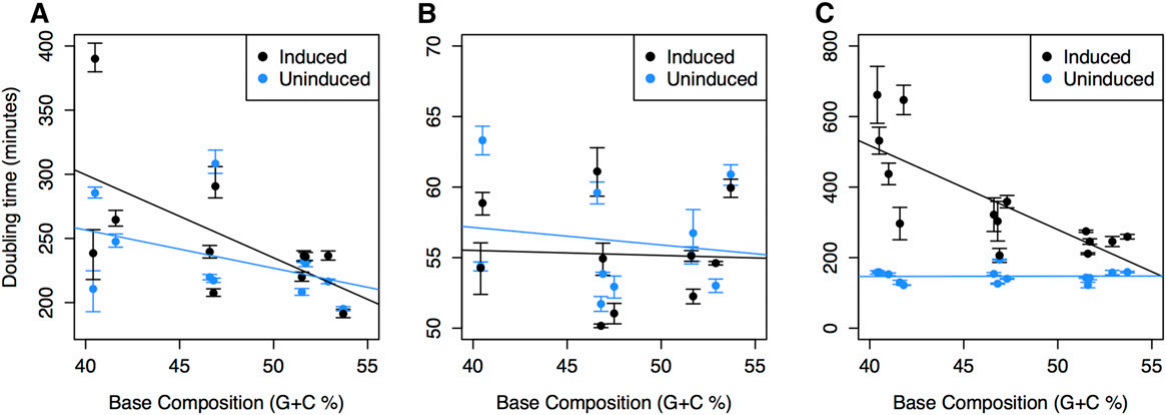
Relationship between base composition of GFP genes and doubling times in hosts expressing GFP gene variants having different base composition at synonymous sites: (A) *C. crescentus*; (B) *P. aeruginosa*; and (C) *E. coli*. A significant positive association between G+C content and fitness was observed for *C. crescentus* (*P* = 0.04; *r^2^* = 0.30) and *E. coli* (*P* = 0.001; *r^2^* = 0.55) on induction of GFP expression (black circles); no such significant relationship was observed in *P. aeruginosa* (*P* = 0.90) or in any species when GFP was not expressed (blue circles) (*P* = 0.18, *P* = 0.67, and *P* = 0.94 for un-induced cultures of *C. crescentus*, *P. aeruginosa*, and *E. coli*, respectively). Error bars designate the SEM, which represents how well the sample mean represents the corresponding population mean.

The tested strain CB15 (NA1000) has a base composition of 90% at four-fold degenerate sites (GC4) and 62% G+C at noncoding sites. Assaying the growth rates of isogenic strains that each expressed one of 10 GFP variants having different base compositions at synonymous sites, there is a significant relationship between the G+C content of the expressed GFP gene and growth rate (Figure 1A) (*r^2^* = 0.30; *P* = 0.04). When GFP expression is not induced, there is no significant relationship between %G+C and growth rate (*P* = 0.18), indicating that the fitness effect depends on gene expression and/or protein production. These results are analogous to those previously obtained for *E. coli* (Figure 1C) (Raghavan *et al.* 2012); however, unlike *E. coli*, *C. crescentus* shows a weak negative relationship between G+C content and doubling time even in the absence of the inducer (*P* = 0.1). This difference is likely the consequence of leaky GFP expression in the noninduced cultures of *C. crescentus*, which is higher than that observed in *E. coli* (Figure S1).

**Figure 2:**
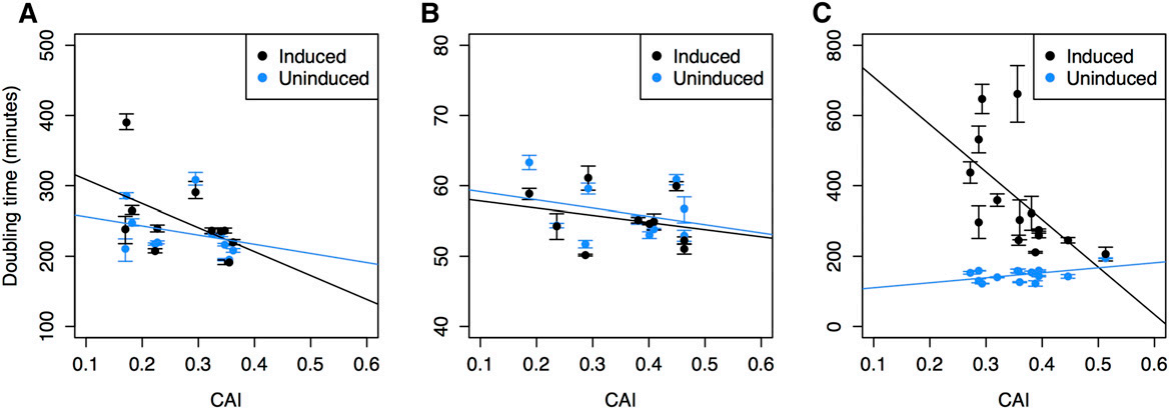
Relationship between codon adaptation indices (CAI) of GFP gene variants and doubling time of (A) *C. crescentus*, (B) *P. aeruginosa*, and (C) *E. coli*. A significant positive association between CAI and fitness was observed in *E. coli* (*P* = 0.03; *r^2^* =0.30) on induction of GFP but no significant relationship was observed when GFP was not expressed (*P* = 0.07). There is no significant relationship between the CAI of GFP genes and doubling times in *P. aeruginosa* under induced (*P* = 0.48) and not induced (*P* = 0.40) conditions. Similarly, in *C. crescentus*, CAI is not significantly related to doubling times under induced (*P* = 0.12) and not induced (*P* = 0.37) conditions. Error bars designate the SEM, which represents how well the sample mean represents the corresponding population mean.

Because codon usage in highly expressed genes can be biased for tRNA optimization (Sharp *et al.* 2005, 2010; Dong *et al.* 1996; Ikemura 1985), we also examined whether the relationship between growth rate and G+C content of the GFP variants might be attributable to codon usage preferences, as indicated by the CAI. The relationship between CAI of the GFP gene variant and bacterial fitness is not significant in *C. crescentus* (*P* = 0.1) (Figure 2A), which differs from the situation for *E. coli*, whereby some of the GFP gene variants yielding higher growth rates show bias toward those codons observed in highly expressed genes (*P* < 0.05) (Figure 2C) (Raghavan *et al.* 2012).

Because the effect of G+C contents at synonymous sites in *C. crescentus* is not associated with the tRNA optimization, we then examined whether the amount of protein produced by the different GFP variants might be responsible for differences in growth rates. We found that bacterial growth rates are independent of GFP protein production, as measured using GFP fluorescence (*P* = 0.2) (Figure S2).

The presence of SD-like sequences in transcripts is associated with translational pausing in bacteria because such sequences have high affinity to the anti-SD sites of bacterial ribosomes (Li *et al.* 2012). As a result, SD-like sequences are disfavored in bacterial coding regions. We examined whether the fitness variation in our bacterial strains could be explained by differences in the frequency of SD-like sequences in the GFP variants. For each GFP variant, we counted the frequency of those SD-like sequences that have high, predicted affinity for the ribosomsal anti-SD sites (>6 kcal/mol) (Li *et al.* 2012). We found that the frequency of anti-SD sites was not significantly associated with fitness of *C. crescentus* strains (*P* = 0.381). We also noted a similar absence of relationship between frequency of SD-like sites and fitness in the isogenic *E. coli* strains (*P* = 0.16) (Raghavan *et al.* 2012). This suggests that selection on G+C-content of bacterial genomes is not driven by avoidance of SD-like sequences in coding regions.

### Pseudomonas aeruginosa

The tested strain, PA01, has a base composition of 91% G+C at four-fold degenerate sites (GC4) but an expected GC4 of only 71% based on its mutational spectrum (Hildebrand *et al.* 2010), again implying a role for selection in increasing the G+C contents of protein-coding genes. In contrast to the situation for *C. crescentus* and in *E. coli* (Raghavan *et al.* 2012), we detected no significant relationship between the G+C contents of the expressed GFP gene variants and growth rate in this strain (Figure 1B) (*P* = 0.90). Additionally, and again unlike the situation for *C. crescentus* and in *E. coli*, reporter gene expression results cause little change in the growth rate of *P. aeruginosa*. The maximum difference in doubling time between induced and noninduced strains of *P. aeruginosa* was only 1.08-fold, as opposed to 5.3-fold and 1.4-fold for *E. coli* and *C. crescentus*, respectively. Moreover, the maximal difference in doubling times among strains in which different GFP gene variants were induced was only 1.2-fold for *P. aeruginosa* compared to 3.2-fold and 2.0-fold for *E. coli* and *C. crescentus*, respectively. Together, these findings show that the expression and translation of GFP pose little metabolic burden on *P. aeruginosa* in that this reporter gene system is insensitive to fitness effects that might be associated with changes in base composition in this species.

## Discussion

The variation in genomic base composition among bacteria has been thought to arise primarily from species-specific differences in the frequencies of each mutation (Sueoka 1962; Freese 1962). Recent comparisons of closely related genomes indicate that, for most genomes, the input of new mutations would not produce the observed base compositions. In general, new mutations would almost universally result in genomes that are more A+T-rich. The disparity between the G+C content expected from new mutations to a genome and its current base composition is best explained by the action of natural selection or by another process, such as biased gene conversion. Naturally, missense and nonsense mutations are affected by selection on protein structure and function, which might serve to alter overall base composition of genes (although not necessarily toward higher G+C); however, genome comparisons have shown that selection for increased G+C contents also acts on "silent" sites, *i.e.*, noncoding DNA and synonymous codon positions, previously assumed to evolve in a neutral manner. We adopted an experimental approach to study the effects of selection for increased G+C contents and to gain some understanding about the substrate on which selection is operating.

### Two types of codon usage bias operating in bacterial genomes

We tested whether the expression of gene variants differing only in their base compositions at synonymous sites exerted differential effects on the cellular growth rates of two G+C-rich bacteria, *C. cresentus* and *P. aeruginosa*. In *C. cresentus*, the strains expressing G+C-rich gene variants had higher growth rates than strains producing the identical protein from G+C-poor gene variants, mirroring results previously reported for *E. coli* (Raghavan *et al.* 2012). In both *C. cresentus* and *E. coli*, selection favors synonymous codons that were G+C-rich; although there is >30% difference between the G+C contents at four-fold degenerate sites in the two species.

It has long been known that the highly expressed genes in many bacterial genomes utilize a very limited set of the possible synonymous codons, an adaptation thought to optimize the use of available tRNAs, thereby promoting translation speed and/or accuracy (Sharp *et al.* 2005, 2010; Dong *et al.* 1996; Ikemura 1985). Selection for increased G+C at synonymous sites, as detected in our experiments, also represents a form of bias in codon usage, and we can examine the extent to which these two biases—one for tRNA optimization and the other for increased G+C content—overlap and select for the same codons.

Our experiments used identical GFP gene variants in both *E. coli* and *C. cresentus*, but because each species has different codon usage preferences in its highly expressed genes, the association between selection for optimal codons (as reflected by CAI values) and selection for higher G+C codons are not the same in the two species (Figure 2). Although synonymous sites of *C. cresentus* are very G+C-rich, there is no significant association between the CAI of the GFP gene variants and growth rates in this species. In contrast, the relationship between growth rates and the G+C contents of the GFP gene variants in *E. coli* is confounded to some extent by codon biases for tRNA optimization in that the gene variants conferring the highest growth rates contain synonymous codons preferred by highly expressed genes.

The association between growth rate and the CAI of the expressed GFP gene variant was not originally recognized in *E. coli* due to differences in the numbers of genes in the reference sets used to derive CAI values (14 *vs.* 51) (Raghavan *et al.* 2012). To distinguish between the roles of the two types of codon usage preferences (tRNA optimization and increased G+C), we first isolated the relationships of GFP genes’ CAI with G+C content and, separately, with bacterial fitness, using two separate linear regressions, and then examined the relationship between the residual variations in fitness and the G+C contents. Using these procedures to examine each factor separately, we found that *E. coli* growth rates are significantly associated with the overall G+C content of the expressed GFP gene variants (*P* = 0.012; *r^2^* = 0.37), and that CAI is not significantly related to fitness after factoring out the effect of G+C contents on fitness (*P* = 0.39).

These results show that the base composition of a single expressed gene can contribute to cellular growth rates and that in two of the species tested, selection favors increases in the G+C contents of expressed genes. Genomes with very different base compositions and codon usage patterns showing a similar selective response to higher genic G+C contents indicate that synonymous codon choice in bacteria is subject to two adaptive forces and that these forces need not operate in the same direction.

### On what process is selection acting?

Based on several observations, it appears this selective force to increase genic G+C contents is operating during the process of translation. First, differences in growth rates are observed only when the reporter genes are expressed, limiting the action of selection to transcription, translation, or protein efficiency. Second, there is no relationship between genic G+C content and the amount of protein that the corresponding gene produces, implying that the selective differences between G+C-rich and G+C-poor variants are not caused by the overall accumulation of the encoded protein. Third, the relationship between the genic G+C content and growth rates is not apparent after removal of the ribosome-binding site from the gene (Raghavan *et al.* 2012), which excludes transcription as a major substrate for this selection force. Thus, the G+C effect on growth rates might be caused by differences in the ways that A+T-rich and G+C-rich mRNAs interact with the ribosome. This suggests that selection is occurring during the process of translation, likely mediated by the thus far unrecognized effects of G+C contents on translational speed or accuracy, or on mRNA secondary structure and stability.

These results lend support to the hypothesis that selection is responsible for the relative G+C richness of coding regions of multiple bacterial genomes, despite a mutational bias toward A/T (Hershberg and Petrov 2010; Hildebrand *et al.* 2010). These findings also help explain why the G+C content of synonymous sites often differs from that of intergenic regions, particularly in G+C-rich and G+C-moderate genomes, although both are generally considered to evolve neutrally (Raghavan *et al.* 2012). It should be noted that the base composition of intergenic regions, like that at synonymous sites, need not be strictly neutral and reflect the underlying pattern of mutation; intergenic regions often contain functional, noncoding elements whose sequences are under selective constraints and their base compositions might also be modified by a nonselective process, such as biased gene conversion (Duret and Galtier 2009; Touchon *et al.* 2009; Birdsell 2002; Lassalle *et al.* 2015). Given the results of experimental and comparative analyses, it currently appears that genomic base composition stems from a variety of sources rather than from a single selective agent acting on the entire genome.

### How widespread among bacteria is selection for increased G+C contents?

The majority of sequenced bacterial genomes are G+C-rich in their coding regions (Raghavan *et al.* 2012), and we propose that translational selection has played a role in the compositional bias in many of these genomes. However, in contrast to the situation for *C. crescentus* and *E. coli*, we saw no significant relationship between the genic G+C content and growth rates in *Pseudomonas aeruginosa*. Although it is tempting to conclude that selection on genic G+C does not occur in this species, and that the high G+C content of its genome results from other factors, the disparity is most likely attributable to aspects of our experiment system. In *P. aeruginosa*, expression of GFP reporter genes did not impact growth rates, preventing the differentiation of any selective effects caused by the reporter gene variants (whereas in *C. crescentus* and *E. coli*, induction of the reporter gene resulted in a reduction in doubling times of up to five-fold). It is possible that the relatively low base composition of the pNW33N plasmid used for reporter gene expression in *P. aeruginosa* precludes a sufficiently high level of GFP expression to detect a selective effect. It is notable, however, that *P. aeruginosa*, unlike most other bacteria with high G+C contents and relatively large genome sizes, displays weak codon usage bias in its highly expressed genes relative to its moderately expressed genes (Sharp *et al.* 2010), raising the possibility that additional forces contribute to base composition in some of the most G+C-rich bacterial genomes.

We focused primarily on the G+C-rich bacterial genomes, because these are presumably under the strongest selection for increased G+C given the mutational bias toward A+T. However, there also exist bacterial taxa that have large effective population sizes and that consist primarily of A+T-rich genomes (Raghavan *et al.* 2012), and we might expect that in such taxa, which include *Bacillus* and *Vibrio*, translational selection on G+C content does not operate. As a group, these genomes do not exhibit as much compositional disparity between noncoding and synonymous coding sites as do the G+C-rich genomes (Raghavan *et al.* 2012).

In conclusion, we found new evidence of selection on the nucleotide composition of expressed protein-coding genes in the evolutionarily and compositionally divergent genomes of *E. coli* and *C. crescentus*: both species display higher growth rates when expressing G+C-rich versions of genes encoding the identical protein. We also observed that our experimental technique cannot effectively test this G+C effect in all bacterial species, and an alternative experimental method may be required for investigating these species. Our results indicate that, in addition to the codon usage preferences that optimize tRNA usage in highly expressed genes, there is selection to increase the G+C contents of protein regions, and that the two forms of selections need not be acting in concert. Our results also suggest that the strength of selection on genic G+C content, like that on codon usage preferences, varies among bacteria and contributes to the wide variation in genomic base compositions.

## Supplementary Material

Supporting Information
