# Supplementary material for: Effects of Genic Base Composition on Growth Rate in G+C-rich Genomes
Source: G3 (Bethesda). 2015 Apr 20;5(6):1247–52. doi: 10.1534/g3.115.016824 (PMC4478552; doi:10.1534/g3.115.016824)
Supplement: Supporting Information [file supp_5_6_1247__index.html]

Effects of Genic Base Composition on Growth Rate in G+C-rich Genomes — Supporting Information 

# Effects of Genic Base Composition on Growth Rate in G+C-rich Genomes

## Supporting Information for Kelkar, Phillips, and Ochman, 2015

**Files in this Data Supplement:**

- Supporting Information - Figures S1-S2 and Tables S1-S3 (PDF, 241 KB)
- Figure S1 - Difference in normalized fluorescence between induced (black circles) and non-induced (blue triangles) cultures of: (A) *Caulobacter crescentus*, (B) *Pseudomonas aeruginosa*, and (C) *Escherichia coli*. (PDF, 341 KB)
- Figure S2 - Relationship between GFP production and doubling time in *C. crescentus* strains expressing GFP gene-variants of different base composition at synonymous sites. (PDF, 187 KB)
- Table S1 - Primer sequences and their applications. (PDF, 108 KB)
- Table S2 - Fluorescence and absorbance readings of induced and uninduced cultures of *Caulobacter crescentus*. (.xls, 44 KB)
- Table S3 - Fluorescence and absorbance readings of induced and uninduced cultures of *Pseudomonas aeruginosa*. (.xlsx, 56 KB)
