# Supplementary material for: Effects of Genic Base Composition on Growth Rate in G+C-rich Genomes
Source: G3 (Bethesda). 2015 Apr 20;5(6):1247–52. doi: 10.1534/g3.115.016824 (PMC4478552; doi:10.1534/g3.115.016824)
Supplement: Supporting Information [file supp_g3.115.016824_016824SI.pdf]

G3· Genes | Genomes | Genetics

Effects of Genic Base Composition on Growth Rate in G+C-rich Genomes

Yogeshwar D. Kelkar[†1], Daniel S. Phillips[†], Howard Ochman[†2]

[†]Department of Ecology and Evolutionary Biology, Yale University, New Haven, CT 06520

[1]Current address: Department of Biostatistics and Computational Biology, University of Rochester School of Medicine and Dentistry, Rochester, New York, USA;

[2]Current address: Department of Integrative Biology, University of Texas, Austin, Texas, USA

Corresponding author:

Howard Ochman
University of Texas
2506 Speedway, NMS 4.216
Austin, TX 78712
Email: howard.ochman@austin.utexas.edu
Phone: 512-232-5666

DOI: 10.1534/g3.115.016824

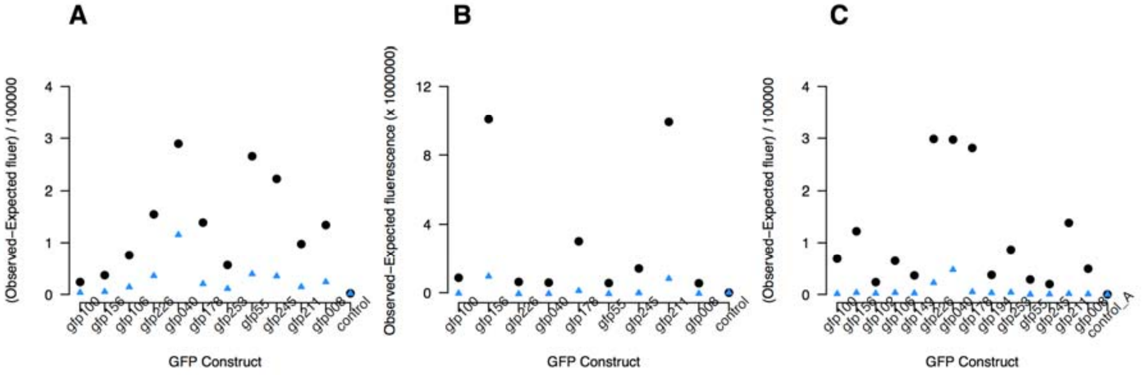

Figure S1 Difference in normalized fluorescence between induced (black circles) and non-induced (blue triangles) cultures of: (**A**) *Caulobacter crescentus*, (**B**) *Pseudomonas aeruginosa*, and (**C**) *Escherichia coli*. Non-induced strains of *C. crescentus* produce greater fluorescence in relation to non-induced strains of *E. coli* and *P. aeruginosa.* Values for *E. coli* were obtained from R. Raghavan, Kelkar YD, Ochman H. 2012. Proc. Natl. Acad. Sci. USA **109**:14504–14507.

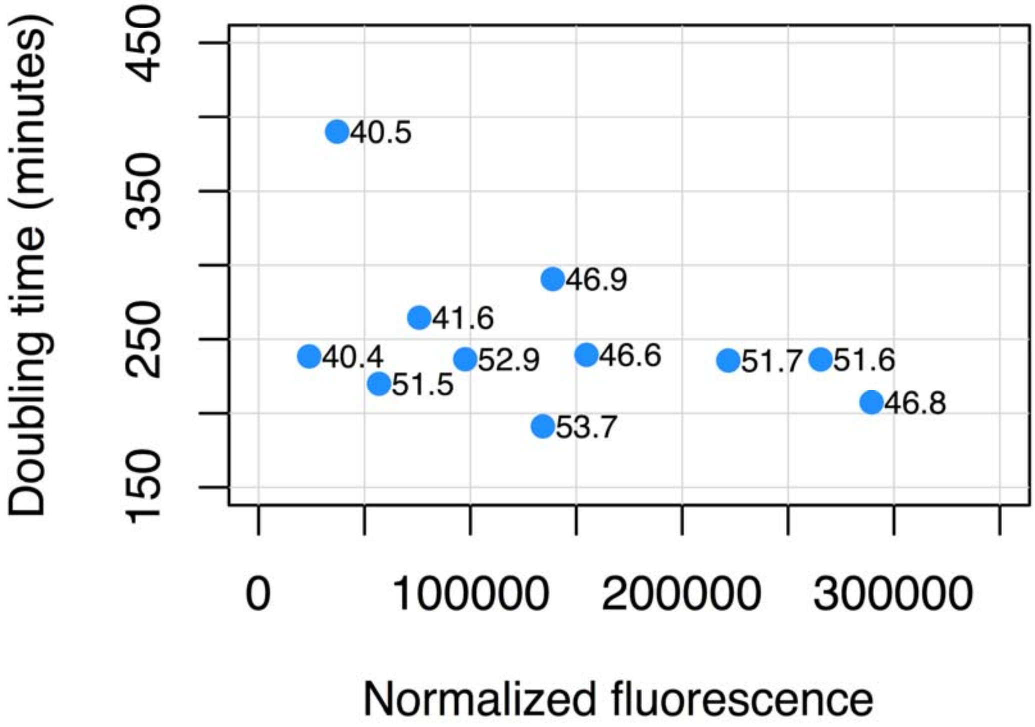

Figure S2 Relationship between GFP production and doubling time in *C. crescentus* strains expressing GFP gene-variants of different base composition at synonymous sites. (Normalized fluorescence of bacterial cultures used as a proxy for GFP expression.) Numbers next to plotted points denote the base composition (G+C%) of the corresponding GFP variant.

Table S1 Primer sequences and their applications

To amplify GFP gene for insertion into:	Primer Name	Sequence (5’ … 3’)
pNW33N	Bpu10I-gfp1	CGACTGGATCCGCTTAGGAGATATAACCATGGTCAG
	Bpu10I-gfp2	CGACTGGATCCGCTTAGGAGATATAACCATGGTTAG
	Bpu10I-gfp3	CGACTGGATCCGCTTAGGAGATATAACCATGGTGAG
	Bpu10I-gfp6	CGACTGGATCCGCTTAGGAGATATAACCATGGTAAG
	BstZ17I-common2	CCTTTCAGCAGTATACCCCTCAAGACCCGTTTAG
pBXMCS-2	Apa1_pet15_F	CAATTCAGTCGACTGGGGCCCAGGAGGAGATATAACCATGG
	EcoR1_pet15_R	CTCCTTTCAGCGAATTCCCCCTCAAGACCCGTTTAG

Tables S2-S3
Available for download as Excel files at www.g3journal.org/lookup/suppl/doi:10.1534/g3.115.016824/-/DC1

Table S2 Fluorescence and absorbance readings of induced and uninduced cultures of *Caulobacter crescentus*.

Table S3 Fluorescence and absorbance readings of induced and uninduced cultures of *Pseudomonas aeruginosa*.
