# Supplementary material for: Effects of Genic Base Composition on Growth Rate in G+C-rich Genomes
Source: G3 (Bethesda). 2015 Apr 20;5(6):1247–52. doi: 10.1534/g3.115.016824 (PMC4478552; doi:10.1534/g3.115.016824)
Supplement: Supporting Information [file supp_g3.115.016824_FigureS2.pdf]

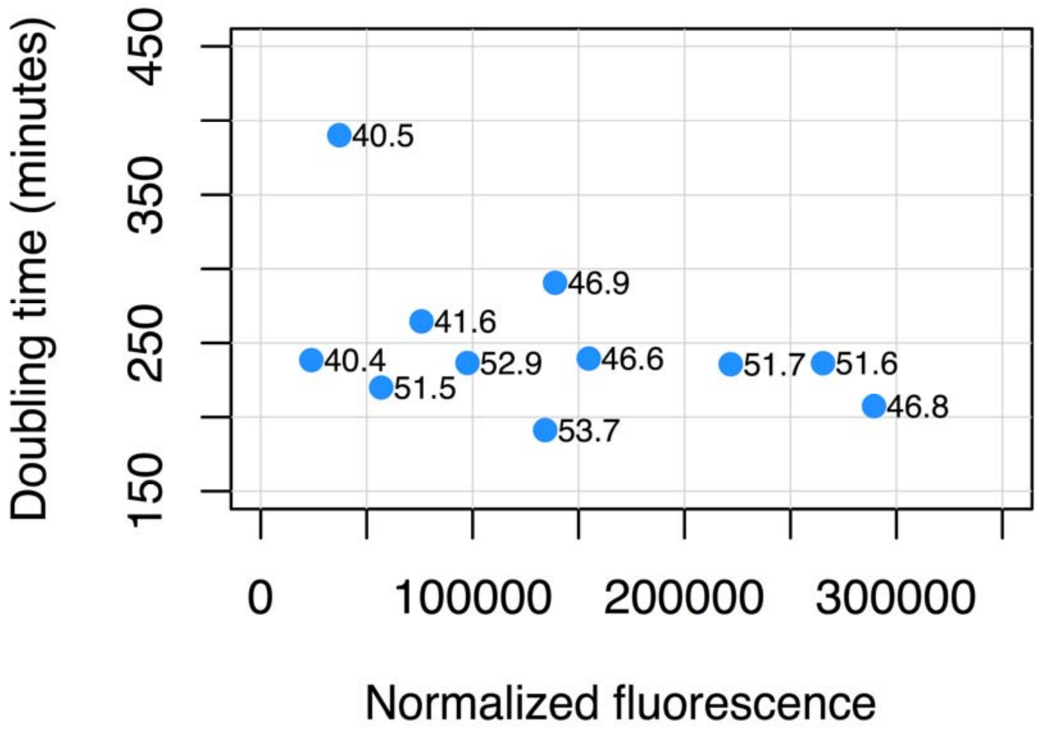

Figure S2 Relationship between GFP production and doubling time in *C. crescentus* strains expressing GFP gene-variants of different base composition at synonymous sites. (Normalized fluorescence of bacterial cultures used as a proxy for GFP expression.) Numbers next to plotted points denote the base composition (G+C%) of the corresponding GFP variant.
