# Supplementary material for: Effects of Genic Base Composition on Growth Rate in G+C-rich Genomes
Source: G3 (Bethesda). 2015 Apr 20;5(6):1247–52. doi: 10.1534/g3.115.016824 (PMC4478552; doi:10.1534/g3.115.016824)
Supplement: Supporting Information [file supp_g3.115.016824_TableS1.pdf]

Table S1 Primer sequences and their applications

To amplify GFP gene for insertion into:	Primer Name	Sequence (5’ … 3’)
pNW33N	Bpu10I-gfp1	CGACTGGATCCGCTTAGGAGATATAACCATGGTCAG
	Bpu10I-gfp2	CGACTGGATCCGCTTAGGAGATATAACCATGGTTAG
	Bpu10I-gfp3	CGACTGGATCCGCTTAGGAGATATAACCATGGTGAG
	Bpu10I-gfp6	CGACTGGATCCGCTTAGGAGATATAACCATGGTAAG
	BstZ17I-common2	CCTTTCAGCAGTATACCCCTCAAGACCCGTTTAG
pBXMCS-2	Apa1_pet15_F	CAATTCAGTCGACTGGGGCCCAGGAGGAGATATAACCATGG
	EcoR1_pet15_R	CTCCTTTCAGCGAATTCCCCCTCAAGACCCGTTTAG
