# Supplementary material for: Effects of Genic Base Composition on Growth Rate in G+C-rich Genomes
Source: G3 (Bethesda). 2015 Apr 20;5(6):1247–52. doi: 10.1534/g3.115.016824 (PMC4478552; doi:10.1534/g3.115.016824)
Supplement: Supporting Information [file supp_g3.115.016824_FigureS1.pdf]

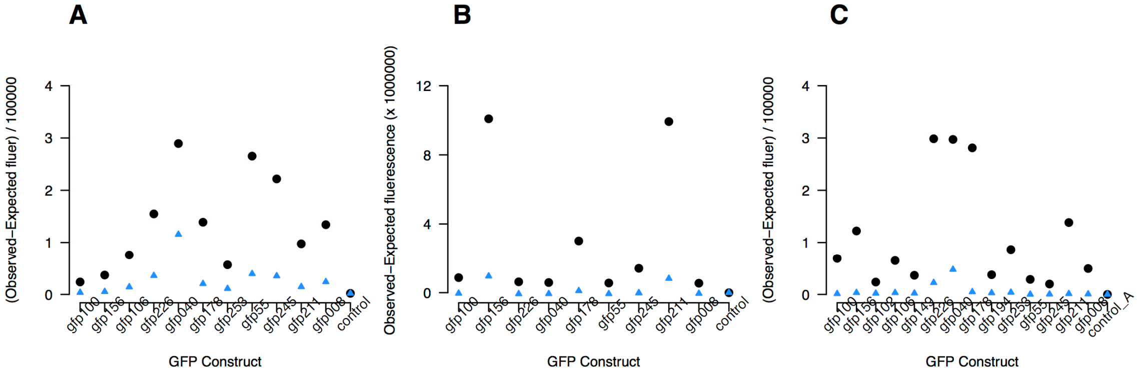

Figure S1 Difference in normalized fluorescence between induced (black circles) and non-induced (blue triangles) cultures of: (**A**) *Caulobacter crescentus*, (**B**) *Pseudomonas aeruginosa*, and (**C**) *Escherichia coli*. Non-induced strains of *C. crescentus* produce greater fluorescence in relation to non-induced strains of *E. coli* and *P. aeruginosa.* Values for *E. coli* were obtained from R. Raghavan, Kelkar YD, Ochman H. 2012. Proc. Natl. Acad. Sci. USA **109**:14504–14507.
